# Antitumor Activity of Emodin against Pancreatic Cancer Depends on Its Dual Role: Promotion of Apoptosis and Suppression of Angiogenesis

**DOI:** 10.1371/journal.pone.0042146

**Published:** 2012-08-02

**Authors:** Sheng-Zhang Lin, Wei-Tian Wei, Hui Chen, Kang-Jie Chen, Hong-Fei Tong, Zhao-Hong Wang, Zhong-Lin Ni, Hai-Bin Liu, Hong-Chun Guo, Dian-Lei Liu

**Affiliations:** 1 Department of Hepato-biliary-pancreatic Surgery, First Affiliated Hospital, Zhejiang University School of Medicine, Hangzhou, China; 2 Department of Oncological Surgery, Zhejiang Cancer Hospital, Hangzhou, China; 3 Department of Hepato-biliary-pancreatic Surgery, Second Affiliated Hospital of Wenzhou Medical College, Zhejiang, China; Faculté de médecine de Nantes, France

## Abstract

**Background:**

Emodin has been showed to induce apoptosis of pancreatic cancer cells and inhibit tumor growth in our previous studies. This study was designed to investigate whether emodin could inhibit the angiogenesis of pancreatic cancer tissues and its mechanism.

**Methodology/Principal Finding:**

In accordance with our previous study, emodin inhibited pancreatic cancer cell growth, induced apoptosis, and enhanced the anti-tumor effect of gemcitabine on pancreatic caner cells *in vitro* and *in vivo* by inhibiting the activity of NF-κB. Here, for the first time, we demonstrated that emodin inhibited tumor angiogenesis *in vitro* and in implanted pancreatic cancer tissues, decreased the expression of angiogenesis-associated factors (NF-κB and its regulated factors VEGF, MMP-2, MMP-9, and eNOS), and reduced eNOS phosphorylation, as evidenced by both immunohistochemistry and western blot analysis of implanted tumors. In addition, we found that emodin had no effect on VEGFR expression *in vivo*.

**Conclusions/Significance:**

Our results suggested that emodin has potential anti-tumor effect on pancreatic cancer via its dual role in the promotion of apoptosis and suppression of angiogenesis, probably through regulating the expression of NF-κB and NF-κB-regulated angiogenesis-associated factors.

## Introduction

Currently, treatment of pancreatic cancer is dependent on surgical resection. However, only about 25% of patients with pancreatic cancer are feasible for surgical resection due to the invasion of pancreatic cancer. Many patients with locally advanced or metastatic pancreatic cancer rely upon gemcitabine chemotherapy [1]. However, pancreatic cancer is a representative of the most highly vascularized and angiogenic solid tumors, which responds poorly to gemcitabine chemotherapy. Previous studies have suggested that treatment with gemcitabine results in the median survival time of about 6.2 months [2]. Thus, seeking new treatment strategies aiming to improve the anti-angiogenic capability is urgent in clinical practice in order to enhance the therapeutic efficacy and inhibit metastasis of pancreatic cancer.

Constitutive activation of nuclear factor-κB (NF-κB) can promote cell proliferation, inhibit cell apoptosis, and regulate the expression of genes associated with angiogenesis [3], [4]. In addition, accumulating evidences suggest that NF-κB plays a major role in the growth, apoptosis inhibition, and angiogenesis of pancreatic cancer [5]. Gemcitabine can enhance the expression and activation of NF-κB in pancreatic cancer cells [6], which is associated with the development of chemoresistance of pancreatic cancer. Therefore, a new drug that inhibits the NF-κB expression and activation may induce apoptosis of cancel cells and reduce the angiogenesis of pancreatic cancer, thus enhancing the anti-tumor activity of gemcitabine and potentially benefiting patients with pancreatic cancer.

Emodin (1,3,8-trihydroxy-6-methylanthraquinone), a natural anthraquinone derivative isolated from Rheum palmatum L, is able to inhibit the growth of pancreatic cancer cells as evidenced by previous studies [6], [7], [8]. Strikingly, it has been reported that emodin has the potential to inhibit several angiogenic processes [9], [10]. It has been suggested that emodin may inhibit cancer cell growth by blocking vascular endothelial growth factor receptor (VEGFR) signaling in human colon cancer cells, indicating that emodin can be used as a potential inhibitor for tumor angiogenesis [11]. However, it remains unclear whether emodin could inhibit the angiogenesis of pancreatic cancer. Interestingly, it has been found that emodin can inhibit the expression and activation of NF-κB [6], [12]. Therefore, in the present study, we aimed to investigate whether emodin can inhibit the growth of pancreatic cancer cells and the angiogenesis of pancreatic cancer via a possible NF-κB involved mechanism. We found that treatment with emodin enhanced the gemcitabine-induced growth inhibition and apoptosis of pancreatic cancer cells *in vitro*. We further demonstrated the tumor growth inhibition and anti-angiogenesis effects of emodin on pancreatic cancer *in vivo*, with downregulating the expression of NF-κB and NF-κB-regulated proteins (i.e. VEGF, MMP-2, MMP-9, and eNOS) and with roles in angiogenesis inhibition and reducing eNOS phosphorylation.

## Results

### Cytotoxicity of Emodin against SW1990 Cells, Panc-1 Cells, HPNE and ECs

**Figure 1:**
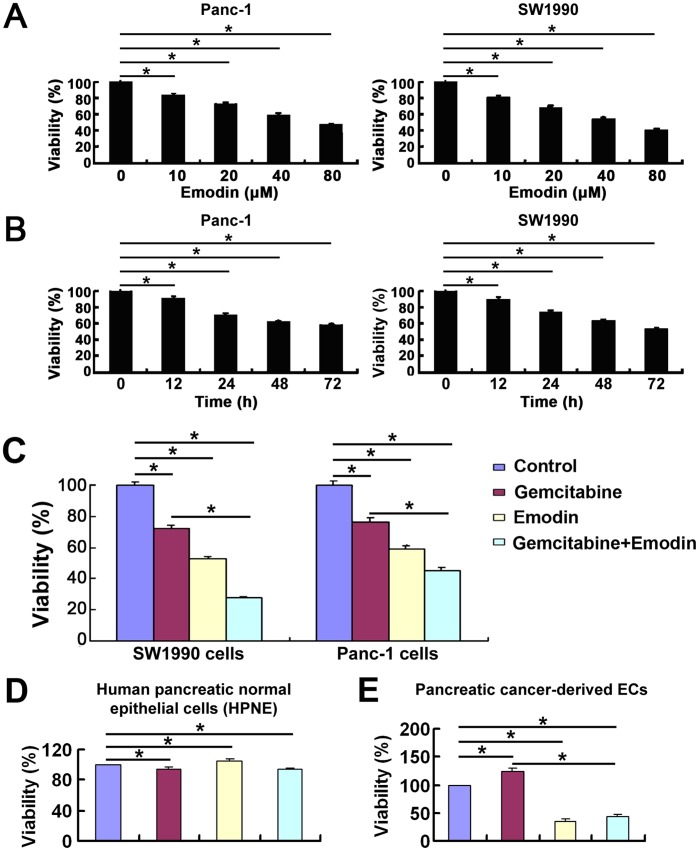
Effect of emodin on SW1990 cells, Panc-1 cells, HPNE and Pancreatic cancer-derived ECs in vitro. The cells without drug treatment were used as the control. (A) Emodin inhibited the proliferation of SW1990 cells and Panc-1 cells in a dose-dependent manner. (B) Emodin inhibited the proliferation of SW1990 cells and Panc-1 cells in a time-dependent manner. (C) Emodin enhanced the proliferation inhibition of SW1990 cells and Panc-1 cells by gemcitabine. (D, E) Viability of HPNE and ECs was assessed after treated with diluent control, gemcitabine (20 µmol/L), emodin (40 µmol/L), or their combination for 72 h. Quantification was performed by calculating the ratio of a value to the control. Data are expressed as mean ± SD of each group of cells from three separate experiments. Emo: emodin; Gem: gemcitabine. *, *P*<0.05.

To test the cytotoxicity of emodin, SW1990 cells and Panc-1 cells were treated with different doses of emodin (10 µM, 20 µM, 40 µM, and 80 µM) for 72 h, and the viabilities of these cells were determined using the CCK-8 assay. As shown in Fig. 1A, treatment with emodin alone reduced the cell viability of both SW1990 cells and Panc-1 cells in a dose-dependent manner. CCK-8 analysis revealed that after SW1990 cells and Panc-1 cells were treated with emodin (40 µM) for 12 h, 24 h, 48 h and 72 h, emodin reduced the cell viability of both cell lines in a time-dependent manner (Fig. 1B). Interestingly, emodin (40 µM) treatment for 72 h reduced the cell viability of SW1990 cells and Panc-1 cells by 46.4% and 40.8%, respectively. However, the combined treatment of both emodin and gemcitabine significantly reduced the cell viability of SW1990 cells and Panc-1 cells by 72.4% and 54.7%, respectively, indicating an enhanced cytotoxicity of the combined drug treatment against SW1990 cells and Panc-1 cells compared to single agent therapy (Fig. 1C) (*P*<0.05). Furthermore, we found that emodin, gemcitabine, and their combination did not change the growth of Human pancreatic normal epithelial cells (HPNE) (Fig. 1D). We found that the gemcitabine (20 µmol/L) treatment significantly reduced cell death in pancreatic cancer-derived ECs, while the emodin (40 µmol/L) treatment and the combined treatment of emodin and gemcitabine enhanced cell death in these cells, indicating that emodin has an inhibitory effect on the proliferation of pancreatic cancer-derived ECs.

### Effect of Emodin on Apoptosis of SW1990 Cells and Panc-1 Cells

**Figure 2:**
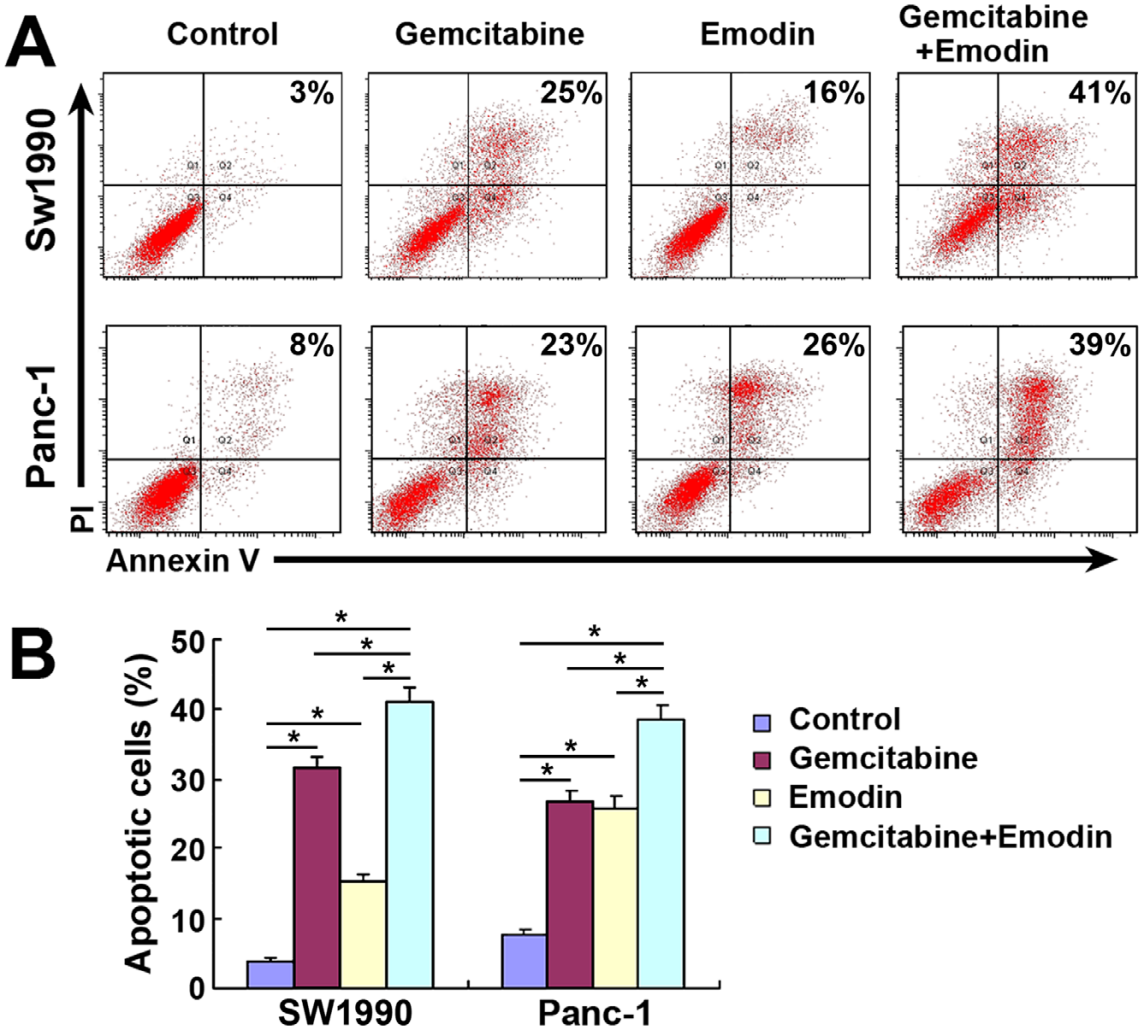
Effect of emodin on the apoptosis of pancreatic cancer cells. (A) Representative dot-plots illustrating the apoptotic status of SW1990 cells and Panc-1 cells. (B) The percentage of apoptotic SW1990 cells and Panc-1 cells. *, *P*<0.05.

Further flow cytometry analysis revealed that treatment with emodin alone increased the apoptosis rate of SW1990 cells from 3.7% to 15.3% and of Panc-1 cells from 7.7% to 26.0%. Compared to single drug treatment, the combined treatment with both drugs induced significantly higher rates of apoptosis (*P*<0.05), which were 41.2% for SW1990 cells and 38.7% for Panc-1 cells (Fig.2A and B). These data are consistent with previous studies of cell growth inhibition using the MTT assay, indicating that the loss of viable cells by emodin and/or gemcitabine is at least partly due to the induction of cell apoptosis.

### Effect of Emodin on Angiogenesis of Pancreatic Cancer-derived ECs

**Figure 3:**
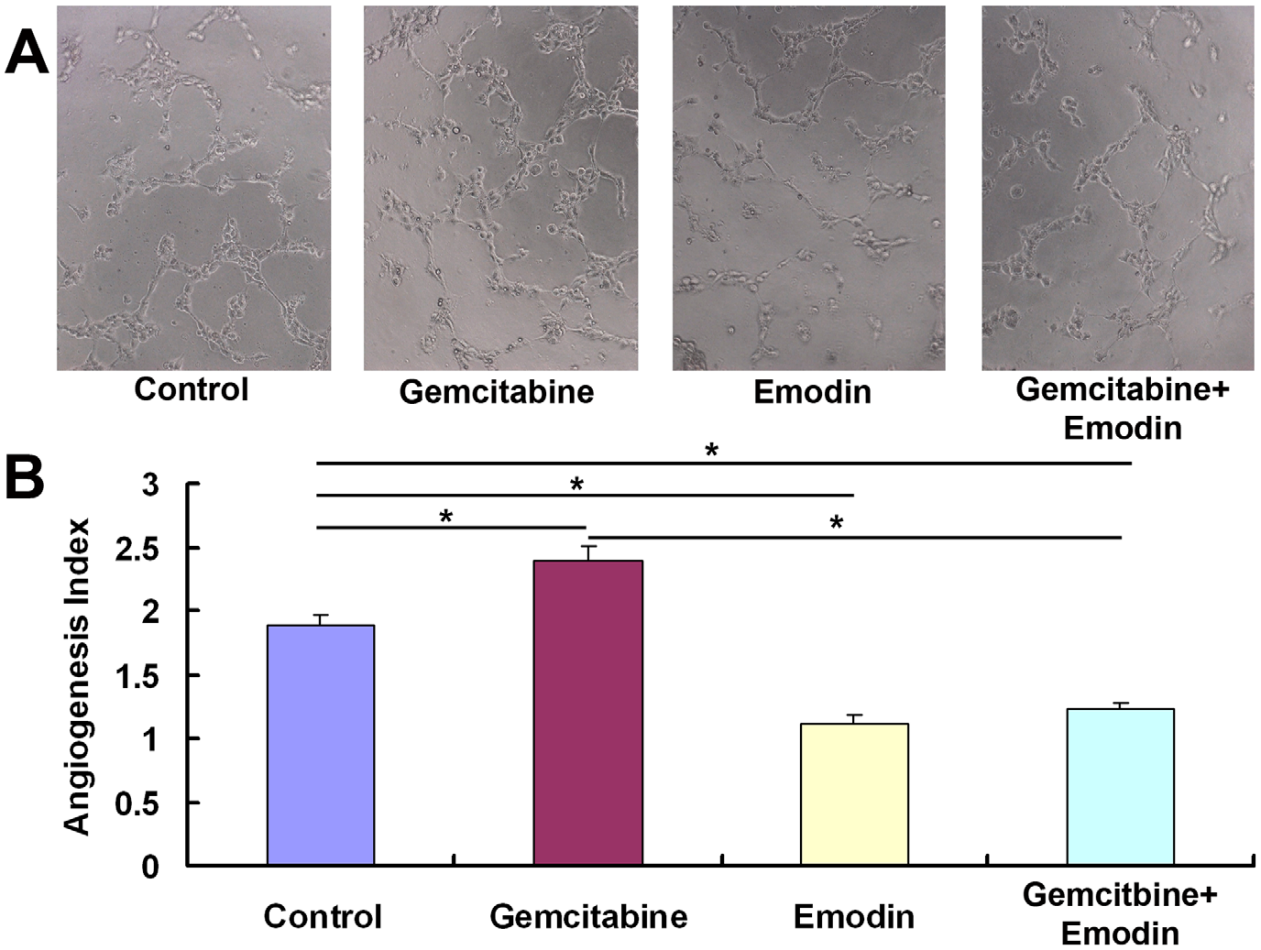
Effect of emodin on the angiogenesis of pancreatic cancer. (A) Representative micrographs (200×) of angiogenesis after treatment of diluent control, gemcitabine (20 µmol/L), emodin (40 µmol/L), or their combination for 72 h. Cells were plated onto the Matrigel-precoated wells (5×10^3^ cells/well) and cultured in 10% FBS-DMEM medium. Original magnification. (B) Angiogenesis of endothelial cells (ECs) isolated from pancreatic cancer tissues. *In vitro* angiogenesis was quantitatively analyzed as described in Materials and Methods. *, *P*<0.05.

The angiogenesis assay was carried out to investigate the effect of emodin on angiogenesis *in vitro*. We found that the gemcitabine (20 µmol/L) treatment up-regulated the index of angiogenesis, while the emodin (40 µmol/L) treatment and the combined treatment of gemcitabine and emodin down-regulated the index, indicating that emodin can inhibit the spontaneous and gemcitabine-induced angiogenesis (Figure 3).

### Effect of Emodin on the Expression and Activity of NF-κB

**Figure 4:**
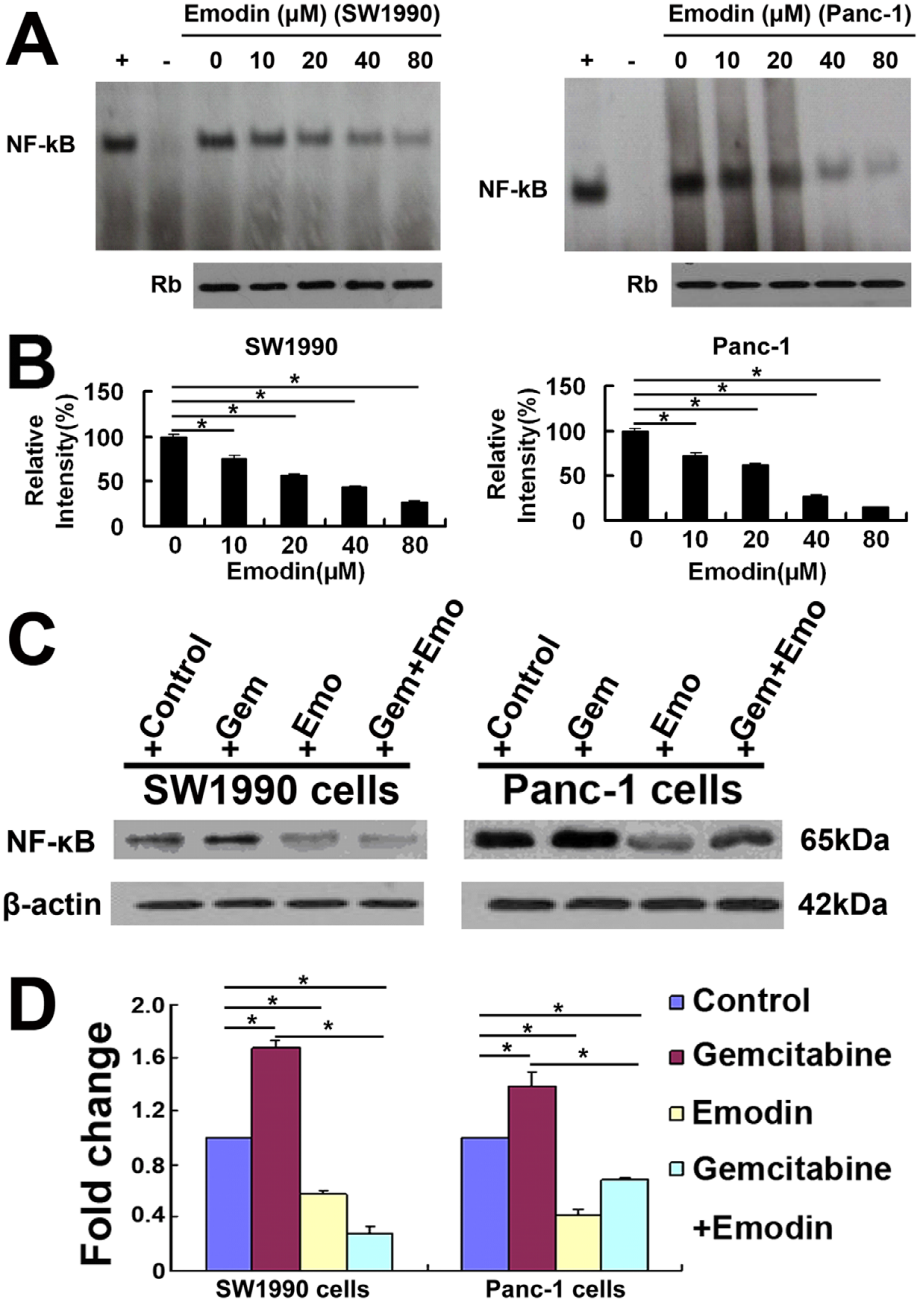
Effect of emodin on the spontaneous and gemcitabine-induced activation of NF-κB *in vitro*. (A) Representative images showing that the treatment with emodin for 72 h inhibited the activity of NF-κB in a dose-dependent manner in SW1990 cells and Panc-1 cells. (B) Data are expressed as mean%±SD of the relative levels of NF-κB activation in SW1990 cells and Panc-1 cells from tree separate experiments. Equal protein loading was ensured by immunoblotting 10 µg of nuclear protein with anti-retinoblastoma antibody. The nuclear extracts from unstimulated gastric cancer SGC7901 cells were used as negative control, and SGC7901 cells stimulated with 50 ng/mL TNFα were used as positive control. +: positive control; –: negative control. (C) Emodin attenuated the spontaneous and gemcitabine-induced NF-κB expression in SW1990 cells and Panc-1 cells. (D) Quantification was performed by calculating the ratio of the value to the control group. *, *P*<0.05.

The results of EMSA analysis showed that treatment with emodin significantly down-regulated the DNA binding activity of NF-κB in SW1990 cells and Panc-1 cells in a dose-dependent manner (Fig. 4A and B). The relative levels of NF-κB/p65 in SW1990 cells and Panc-1 cells were determined using the Western blot assay. We found that gemcitabine up-regulated the expression of NF-κB/p65 in SW1990 cells and Panc-1 cells. However, treatment with emodin alone or combined gemcitabine significantly decreased the spontaneous expression of NF-κB/p65 in both types of cells. NF-κB is a potent transcription factor for regulating the expression of apoptosis-related genes (Fig.4C and D). These data indicated that emodin inhibited the expression of NF-κB and promote pancreatic cancer cell apoptosis *in vitro*, thus enhancing the inhibition of cell growth and proliferation by the combined treatment.

### Effect of Emodin on the Growth of Orthotopically Transplanted Pancreatic Cancer

**Figure 5:**
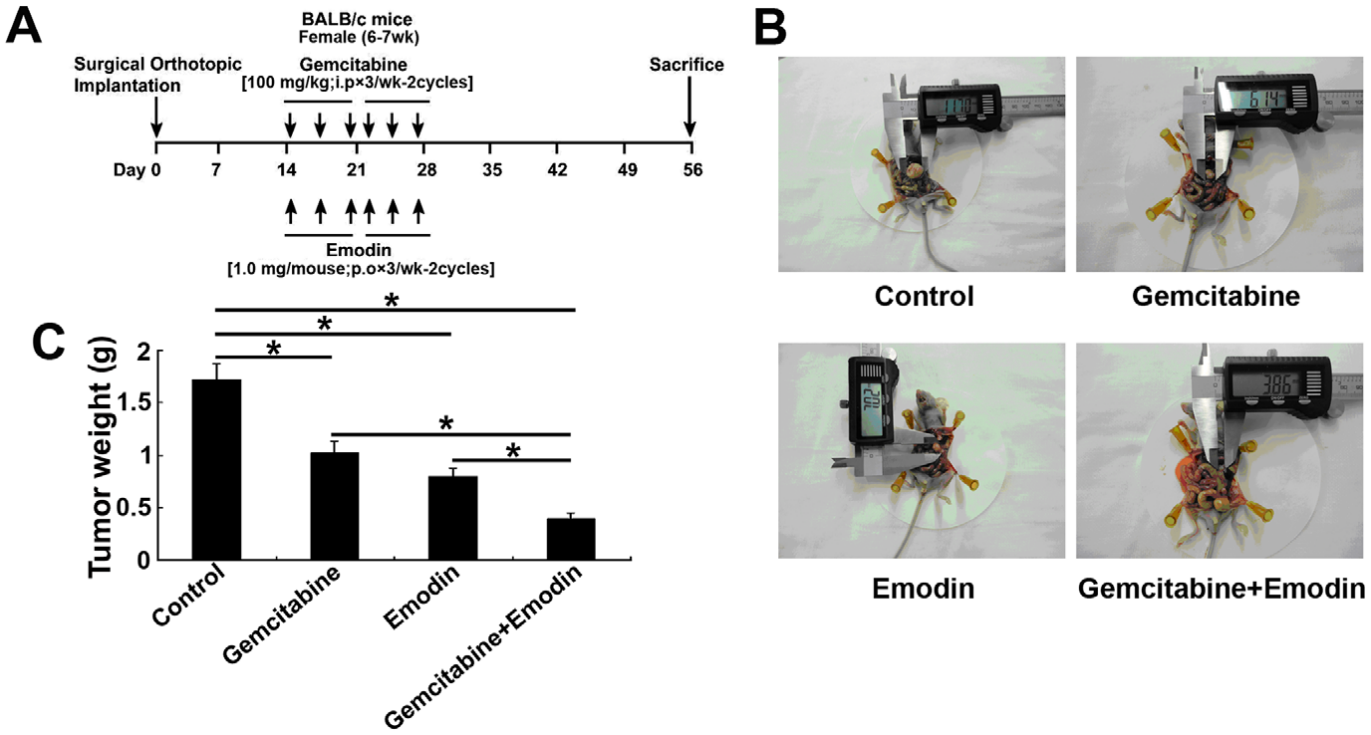
(A) Schematic illustration of experimental protocol. (B) Photographs of orthotopically implanted pancreatic tumors on day 28 following treatment. The combined treatment of emodin and gemcitabine significantly inhibited tumor growth. (C) The tumor weights of individual groups of mice on the last day of the experiment (Day 37). *, *P*<0.05.

The treatment protocol is shown in Fig.5A. At day 56 after tumors were implanted, the average weight of tumors treated with normal saline, emodin (40 mg/kg), gemcitabine (100 mg/kg), and emodin (40 mg/kg) plus gemcitabine (80 mg/kg) was (1.721±0.149) g, (1.021±0.109) g, (0.794±0.082) g, and (0.393±0.057) g, respectively. We observed a significant decrease (*P*<0.05) in tumor weight in the emodin or gemciatabine group compared to the untreated control and the greatest inhibitory effect on tumor growth in the combination group (Fig.5B and C) (*P<*0.05, compared to the control or single drug group).

### Effect of Emodin on the Growth of Orthotopically Implanted Pancreatic Cancer Detected by MicroPET

**Figure 6:**
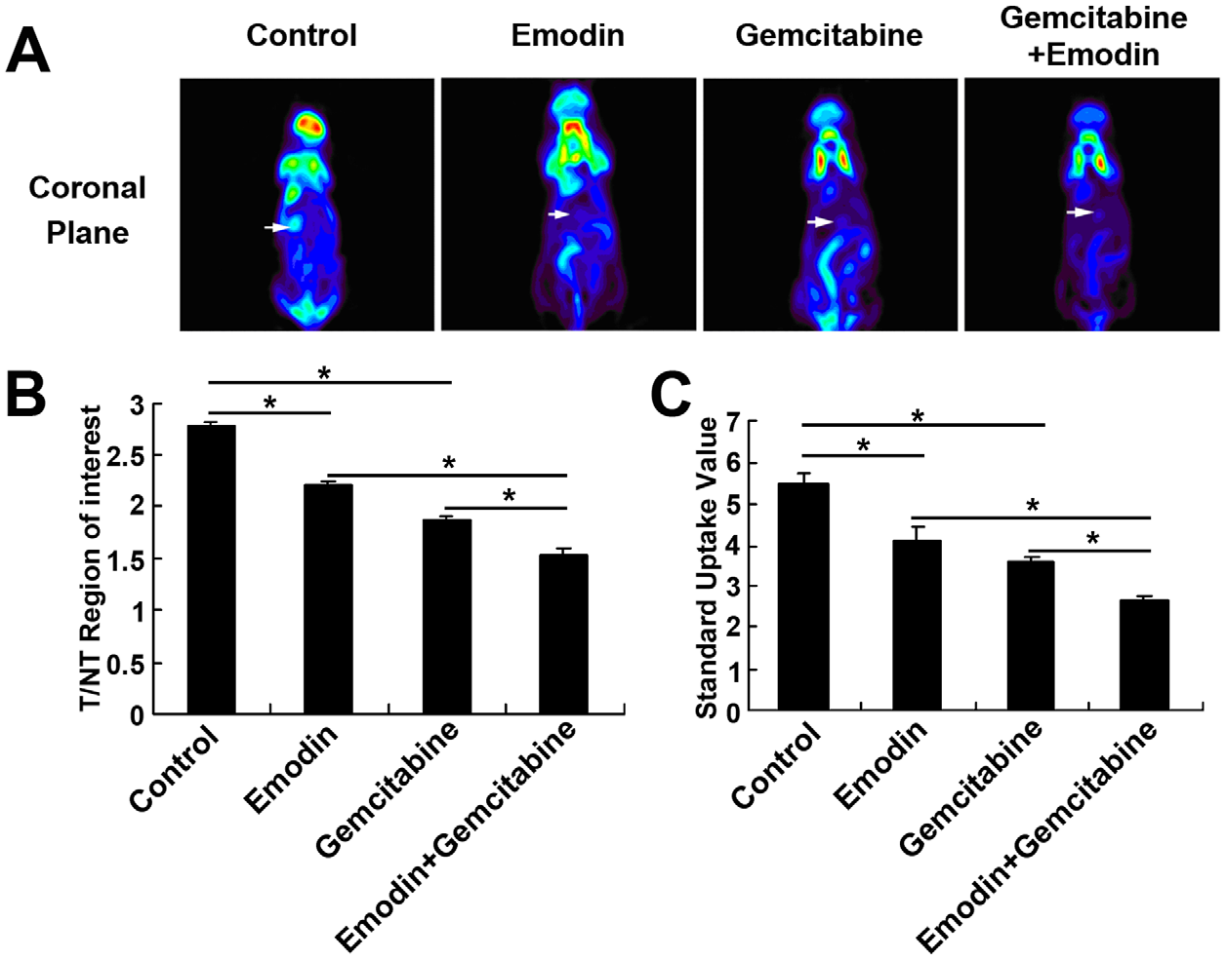
Panc-1 cells were used to establish an orthotopic pancreatic carcinoma xenograft animal model. (A) MicroPET showing a large coronal sectional orthotopic transplantation tumor of a nude mouse. (B and C) T/NT ratio and SUVs in the gemcitabine group, the emodin group and the combination group were lower than that of the control group (0.9% sodium chloride) (*P*<0.05), and the combination group showed even lower T/NT ratio and SUVs than the emodin group and the gemcitabine group. *, *P*<0.05.

After being treated for 4 weeks, the nude mice were imaged with high-resolution positron emission tomography (MicroPET) (Fig. 6A) and fluorine-18-labeled fluorodeoxyglucose (^18^F-FDG) to determine the ratio of tumor to normal muscle tissue (T/NT ratio) in the same size regions of interest (ROI) and standard uptake values (SUV). Compared with the T/NT ratio of the control group (2.78±.042), those of the gemcitabine group (1.88±0.043) and the emodin group (2.20±0.052) were significantly decreased (*P<*0.05), and the combination group (1.55±0.058) was further decreased (*P*<0.05, compared to the single drug groups) (Fig. 6B). The SUVs of the gemcitabine group (3.61±0.12) and the emodin group (4.10±0.35) were significantly decreased compared to the control group (5.49±0.22) (*P<*0.05), and the combination group (2.72±0.08) had even lower uptake than the single drug groups (*P<*0.05) (Fig. 6C).

### Effect of Emodin on Angiogenesis of Orthotopically Implanted Pancreatic Cancer Detected by Tumor Vascular Imaging

**Figure 7:**
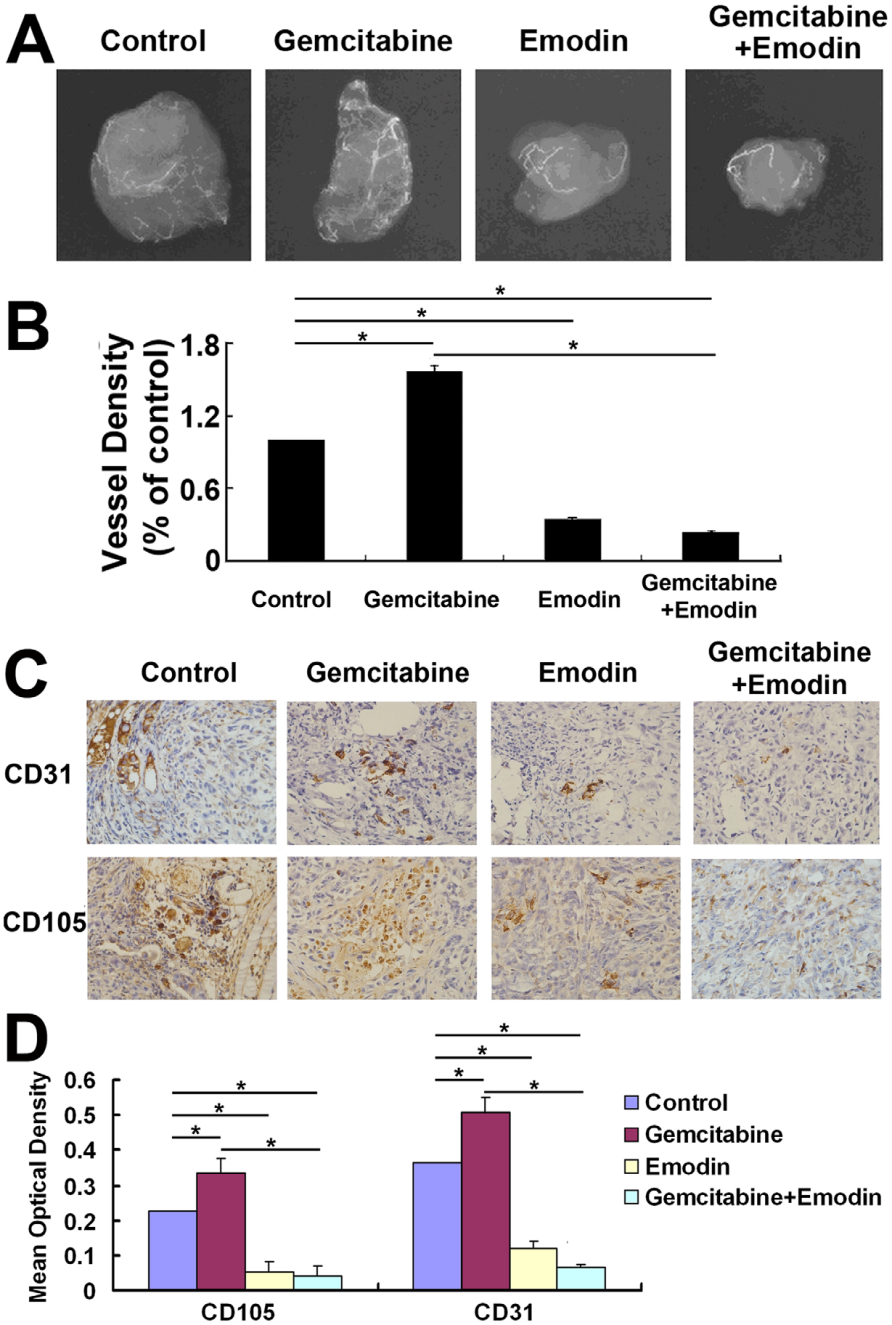
Effect of emodin on the angiogenesis of pancreatic cancer. A: The vascular image of the tumors were captured by X-ray machine. B: Vascular density was calculated as the total tumor blood vessel number divided by tumor volume, and then normalized to the control group. (C) Emodin alone or combined with gemcitabine inhibited the expression of CD31 and CD105. (D) Quantified data are presented. The quantification was performed by calculating the ratio of the value to the control group. *, *P*<0.05.

As demonstrated in Fig.7A and B, tumor vascular imaging analysis revealed that gemcitabine increased the vessel distribution (*P*<0.05), although it decreased the tumor volume compared to the control group as shown by MicroPET (*P*<0.05). However, we found that both the tumor volume and the vessel distribution in the emodin group and the combination group significantly reduced compared to the control and gemcitabine groups (*P*<0.05) (Fig.7A and B). Further, immunohistochemistry analysis showed that the gemcitabine treatment significantly up-regulated the expression levels of CD31 and CD105, which can be markedly decreased by the emodin treatment and the combined treatment of emodin and gemcitabine (Fig.7C and D). These observations suggested that emodin could inhibit the angiogenesis of orthotopically implanted tumors.

### Effect of Emodin on the Expression of Angiogenesis-associated Proteins in Orthotopic Pancreatic Cancer Tissues Detected by Immunohistochemistry and Western Blot

**Figure 8:**
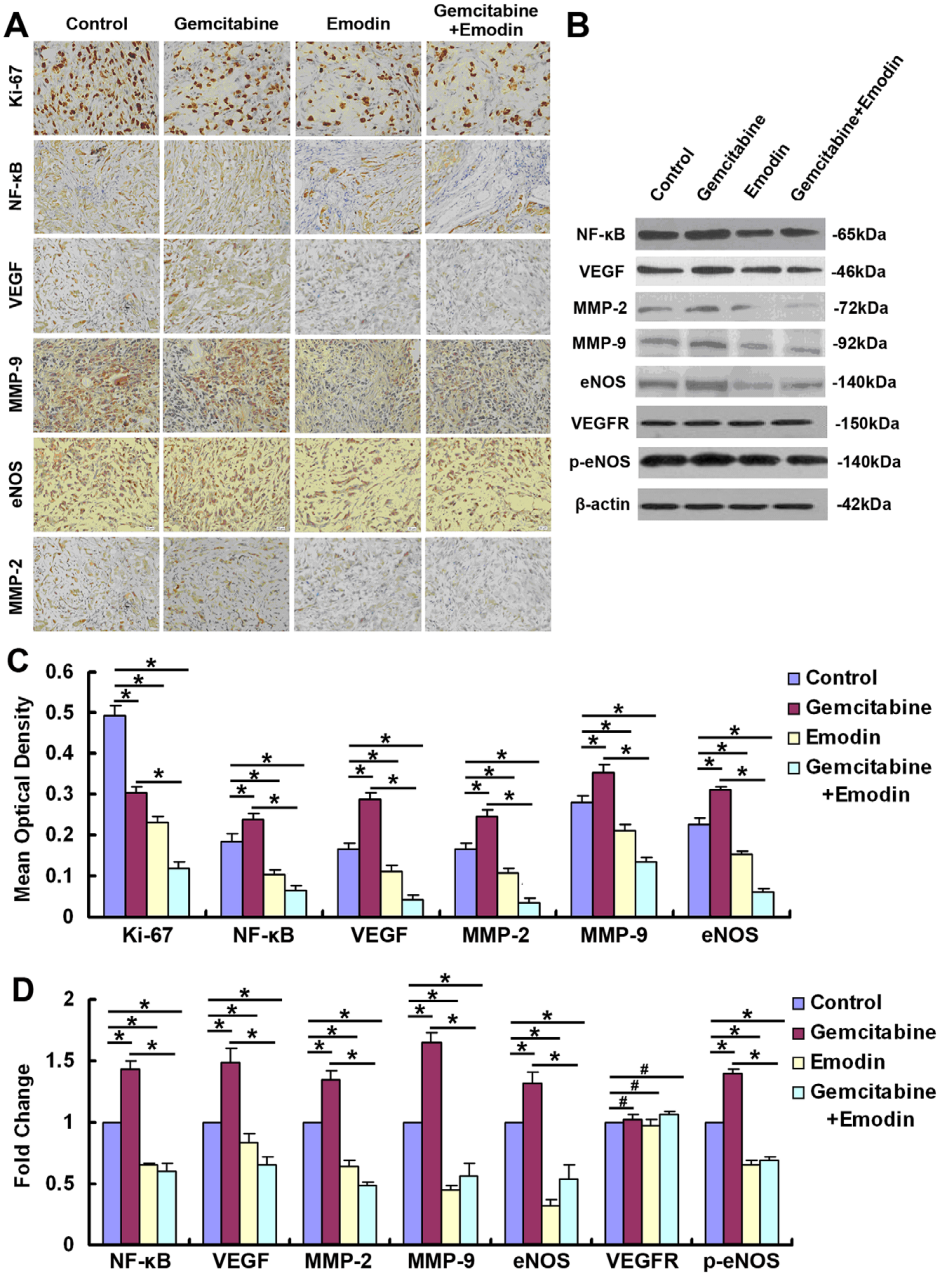
Effect of emodin on the expression of NF-κB and NF-κB-regulated angiogenesis-related proteins (VEGF, MMP-2, MMP-9, and eNOS). (A) Immunohistochemistry analysis of the expression of Ki-67, NF-κB, VEGF, MMP-2, MMP-9, and eNOS in orthotopic pancreatic cancer tissues. (B) Western blot analysis of the expression of NF-κB, VEGF, MMP-2, MMP-9, eNOS, VEGFR, and p-eNOS in orthotopic pancreatic cancer tissues. (C) Quantified data of immunohistochemistry analysis are presented. (D) Quantification of the Western blot results was performed by calculating the ratio of the value to the control group. *, *P*<0.05; #,*P*>0.05.

As shown in Fig. 8, immunohistochemistry analysis revealed that emodin and gemcitabine down-regulated the expression of Ki-67 compared to the control group (*P*<0.05), and their combination further down-regulated the Ki-67 expression compared to the single drug treatments (*P*<0.05). Both immunohistochemistry analysis and Western blot analysis revealed that gemcitabine up-regulated the expression of VEGF, MMP-2, MMP-9, eNOS, and NF-κB in orthotopically thanspanted tumors compared to the control group (*P*<0.05), whereas emodin alone or combined with gemcitabine down-regulated the expression of angiogenesis-associated factors (VEGF, MMP-2, MMP-9, eNOS, and NF-κB) in pancreatic cancer tissues (*P*<0.05). Furthermore, the emodin treatment and the combined treatment of emodin and gemcitabine significantly inhibited eNOS phosphorylation. However, we found that none of the three treatments altered the expression level of VEGFR in pancreatic cancer tissues.

### Effect of Emodin on NF-κB Activity in Transplanted Pancreatic Cancer Tissues

**Figure 9:**
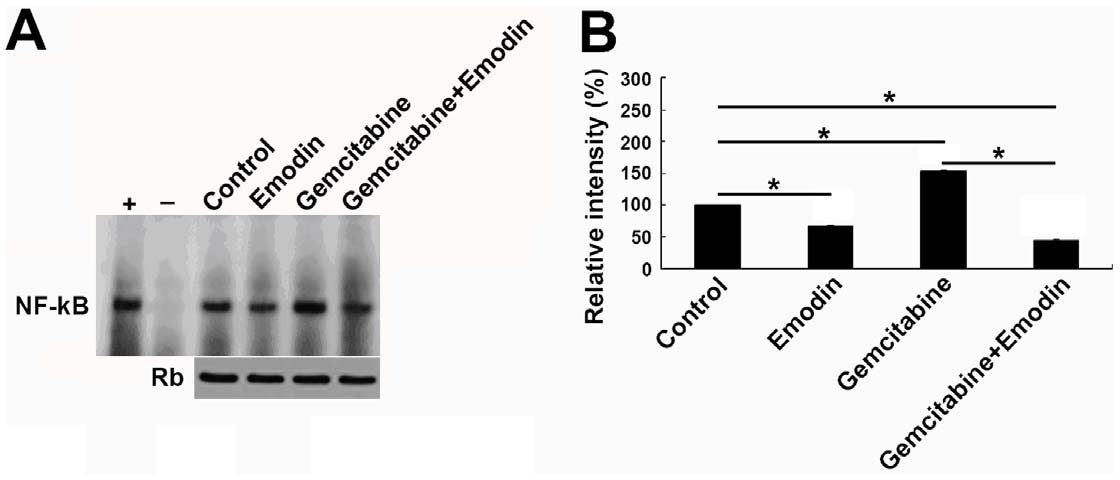
Emodin reduces NF-κB activation. (A) NF-κB DNA binding activity was determined by EMSA and supershift experiment. Equal protein loading was ensured by immunoblotting 10 µg nuclear protein with anti-retinoblastoma antibody. +, positive control; -, negative control. (B) Quantified data are presented. *,*P*<0.05.

To determine whether the combination treatment of emodin and gemcitabine has any effect on NF-κB activation, the DNA binding activity of NF-κB was evaluated using the EMSA assay. Consistent with the immunoblotting results, emodin alone or combined with gemcitabine decreased the DNA binding activity of NF-κB in pancreatic cancer orthotopically implanted with Panc-1 cells (Fig. 9A, B).

### Effect of Emodin on the mRNA Expression Levels of NF-κB, VEGF, MMP-2, MMP-9, and eNOS in Pancreatic Cancer Tissues Detected by RT-PCR

**Figure 10:**
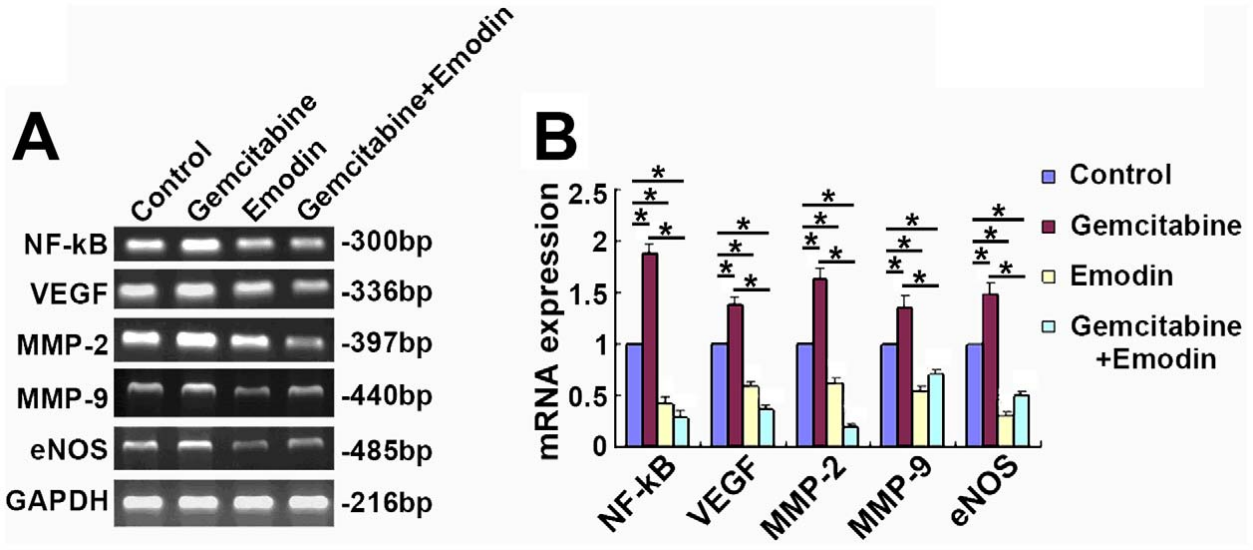
Effect of emodin on the gene expression of NF-κB and NF-κB-regulated angiogenesis-related proteins (VEGF, MMP-2, MMP-9, and eNOS). (A) The mRNA levels of NF-κB, VEGF, MMP-2, MMP-9 and eNOS in pancreatic cancer tissue in different treatment groups were detected by RT-PCR. (B) Quantified data were presented. *, *P*<0.05.

In accordance to the results of immunohistochemistry and Western blot analysis, the mRNA expression of NF-κB, VEGF, MMP-2, MMP-9 and eNOS in pancreatic cancer tissue were up-regulated in the gemcitabine group, but down-regulated in the emodin group and the combination group compared to the control group in pancreatic cancer tissues (*P*<0.05) (Fig. 10A, B).

## Discussion

Consistent with our previous observations [8], the treatment of cells with emodin significantly enhanced the inhibition of cell growth by gemcitabine, which was correlated well with the results of apoptosis analysis. Similarly, we found that emodin inhibited the pancreatic cancer cell growth *in vivo*. Here, for the first time, we found that emodin inhibited the angiogenesis of pancreatic cancer in vivo, downregulated the expression of NF-κB and NF-κB-regulated proteins with roles in angiogenesis inhibition (VEGF, MMP-2, MMP-9, and eNOS). This study was focused on the anti-angiogenesis effect of emodin *in vivo* and the possible mechanism.

It has been reported that gemcitabine induces the activation of NF-κB and subsequently causes drug resistance [13]. Consistent with our previous observations, the present study clearly showed that emodin inhibited the spontaneous activation of NF-κB in a dose-dependent manner in SW1990 cells and Panc-1 cells, and abrogated the gemcitabine-induced NF-κB activation in both types of cells. In addition, we found that emodin alone or combined with gemcitabine presented anti-tumor effects on an orthotopic pancreatic cancer model, as evidenced by a decrease in tumor weight. Similarly, emodin alone or combined gemcitabine has been shown to have potent anti-tumor effect in an subcutaneous tumor model in our previous study [8].

A critical step in angiogenesis involves the local proliferation of endothelial cells. Hee-Jin Kwak et al. demonstrated that emodin effectively inhibits VEGF-A-induced angiogenesis in vitro and in vivo [10]. Alexander D. Crawford et al. reported that emodin inhibited mammalian endothelial cell proliferation and tube formation in vitro [14]. And it has been reported that emodin can inhibit tumor-associated angiogenesis in human colon carcinoma *in vitro* [9]. Our study showed that proliferation of pancreatic cancer-derived ECs and blood vessel formation was inhibited by emodin alone or combined with gemcitabine. Constitutive activation of NF-κB is observed in many cancers including human pancreatic cancer [15]. Strategies targeting the NF-κB regulated pathway may be useful to improve the effects of chemotherapeutic agents such as gemcitabine on tumor-derived endothelial cells [16]. Our study suggested that emodin may inhibit the angiogenesis of pancreatic cancer-derived ECs through NF-κB regulated signaling pathway. Furthermore, the tumor vascular imaging results showed that the vascular density in the emodin group and the emodin/gemcitabine combination group significantly reduced compared to the control and gemcitabine groups. In addition, compared to the control and gemcitabine groups, the emodin treatment and the combined treatment of emodin and gemcitabine significantly reduced the expression levels of CD31 and CD105. Our results suggested that emodin can inhibit the angiogenesis of orthotopically implanted pancreatic cancer.

Earlier studies have suggested that NF-κB can regulate the expression of several angiogenesis-associated proteins such as MMP-2, MMP-9, VEGF, and eNOS [12], [17], [18], [19]. Our previous study has shown that emodin downregulated the activation and expression of NF-κB in SW1990 cell implantation induced pancreatic cancer [20], NF-κB analysis of the orthotopic tumor tissues in this study showed that emodin alone or combined with gemcitabine suppressed NF-κB expression and its activity in pancreatic cancer tissues. Also we found that emodin alone or combined with gemcitabine downregulated the expression of NF-κB-regulated genes (VEGF, eNOS, MMP2 and MMP9), which are closely associated with angiogenesis.

MMP-2 and MMP-9 may be associated with tumor angiogenesis [9], [17]. It has been reported that emodin can inhibit tumor-associated angiogenesis through suppressing MMP-9 expression [9]. In this study, immunohistochemical analysis showed that emodin alone or combined with gemcitabine downregulated the expression of MMP-2 and MMP-9 in orthotopic pancreatic cancer tissues, while gemcitabine had no effect on the expression of MMP-2 and MMP-9. These results suggested that emodin can inhibit the angiogenesis of pancreatic cancer tissues via suppression of MMP-2 and MMP-9.

As an angiogenesis regulatory factor, VEGF is closely associated with angiogenesis [21] and metastasis of pancreatic cancer [22]. As reported by Aggarwal et al., NF-κB-dependent increase of VEGF expression can promote tumor cell survival and vascular angiogenesis [23]. A recent study suggested that emodin can inhibit the expression of VEGF in human neuroblastoma cells [24]. In this study, we found that emodin alone or combined with gemcitabine significantly downregulated the VEGF expression in orthotopically implanted pancreatic cancer. Our results suggested that emodin may inhibit VEGF expression to suppress the angiogenesis of pancreatic cancer. Further experiments demonstrated that emodin, gemcitabine, and their combination had no effect on VEGFR expression, indicating that the inhibition of angiogenesis by emodin in pancreatic cancer was not associated with VEGFR.

eNOS stimulates the release of NO which in turn can promote the proliferation of EPCs [25]. Inhibition of eNOS in human umbilical vein endothelial cells (HUVECs) may inhibit the proliferation and tube formation of endothelial cells [10]. In this study, we found that emodin alone or combined with gemctabine inhibited eNOS expression in orthotopic pancreatic cancer tissues, indicating that inhibition of eNOS expression may be involved in the inhibition of tumor angiogenesis by emodin. Further experiments demonstrated that the emodin treatment and the combined treatment of emodin and gemcitabine significantly inhibited eNOS phosphorylation, indicating that emodin can significantly inhibit the gemcitabine-induced eNOS phosphorylation which is involved in EC migration, proliferation, and tube formation *in vitro* [26].

NF-κB is closely associated with not only apoptosis inhibition, but also angiogenesis of tumors. Our study indicated for the first time that NF-κB may also play an important role in angiogenesis inhibition by emodin in pancreatic cancer *in vivo*, and the enhanced effect of gemcitabine on pancreatic cancer may be achieved through emodin mediated downregulation of the expression of NF-κB and NF-κB-regulated proteins with roles in angiogenesis inhibition (i.e. VEGF, MMP-2, MMP-9 and eNOS). However, how emodin downregulates NF-κB expression is yet to be studied.

## Materials and Methods

### Ethics Statement

The experimental protocols were approved by the Institutional Review Board of First Affiliated Hospital, Zhejiang University School of Medicine.

### Cell Lines and Animals

The human pancreatic cancer cell lines SW1990 and Panc-1 were purchased from American Type Culture Collection. Human pancreatic normal epithelial cells (HPNE) (CHI Scientific INC, Maynard, MA) were stocked in our laboratory. Female athymic BALB/c nu/nu mice (4–6 weeks old) were purchased from Shanghai Cancer Institute for Tumor Implantation. All animals were maintained in a sterile environment within the Animal Experiment Center of Zhejiang University School of Medicine, according to the Laboratory Animal Regulations of the Ministry of Science and Technology of the People’s Republic of China (http://www.most.gov.cn/kytj/kytjzcwj/200411/t20041108_32465.htm).

### Reagents

Emodin was purchased from Sigma (St. Louis, USA), dissolved in dimethylsulfoxide (DMSO) at 0.2 mmol/L in stock and stored at −20°C. The final concentration of DMSO was <0.1%. Gemcitabine was purchased from Ely Lilly (Bad Homburg, Germany) and dissolved in sterile saline at 50 g/L in stock. RPMI-1640 and fetal bovine serum (FBS) were obtained from Gibco BRL (Grand Island, NY, USA). Cell apoptosis reagent kit (Annexin V-FITC and PI) was purchased from Nanjing KeyGen Biotech Co., Ltd (Nanjing, China). Cell counting kit-8 (CCK-8) was purchased from Beyotime Institute of Biotechnology (Haimen, China). Fluorine-18-labeled fluorodeoxyglucose (^18^F-FDG) was provided by Zhejiang University School (Hangzhou, Zhejiang, China). Antibodies were obtained from the following commercial sources: anti-MMP-2, anti-MMP-9, anti-VEGF, and anti-β-actin antibodies were purchased from Epitomics (Burlingame, California, USA); anti-NF-κB, anti-CD31 and anti-CD105 antibodies were purchased from Abcam (Cambridge, MA, USA); anti-eNOS and anti-VEGFR (Flk-1) (C1158) antibodies were purchased from Santa Cruz (Santa Cruz, USA); anti-Phospho-eNOS (Ser1177) antibody was purchased from Cell Signaling (Beverly, MA, USA); anti-Ki-67 antibody and the DAB kit were purchased from Zhongshan Bio-Tech Co., Ltd (Beijing, China). The TRIzol reagent was purchased from Invitrogen (Carlsbad, CA, USA). The RNA fast 200 purification kit was purchased from Fastagen Biotech (Shanghai, China).

Isolation of microvascular endothelial cells (ECs) from implanted pancreatic cancer tissues.

Implanted pancreatic cancer tissues were cut into 1 mm tissue blocks and incubated in 0.1% collagenase I in MEM at 37°C for 2 h. Microvascular ECs were isolated from the tissue blocks using a magnetic bead immunoaffinity endothelial cell isolation kit (Dynal Biotech, Brown Deer, WI). Briefly, Dynabeads M-450 and sheep anti-rat IgG coupled with a monoclonal rat anti-mouse CD31 antibody (30-µL aliquot per 5-ml tube) were incubated in 1 ml of tissue supernatant at 4°C overnight and then washed three times with 10% FBS-DMEM; 1 ml cell suspension was put into the tube containing the washed beads. After incubation with occasional agitation at 4°C for 30 min, the bead-bound cells were recovered, washed, and then digested in 1 ml trypsin/EDTA (GIBCO) to release the beads. The bead-free cells were centrifuged in 10% FBS-RPMI-1640 and then resuspended in 7 ml culture media as described below. The yield of vascular ECs was >98%. The identity of the isolated cells was confirmed by in vitro tube formation in matrigel and staining for VE-cadherin, an exclusive EC marker.

### Cell Culture

HPNE, ECs, SW1990 cells, and Panc-1 cells were cultured in RPMI-1640 culture medium supplemented with 10% heat-incubated FBS (Invitrogen, New York, USA), 100 unit/mL penicillin, and 100 µg/mL streptomycin at 37°C in a humidified 5% CO_2_ atmosphere. Cells were passaged at 70–80% confluence. Luciferase-transfected SW1990 cells were harvested from 70–80% confluent cultures after a brief exposure to 0.25% trypsin supplemented with 0.2% EDTA. Trypsinization was terminated with RPMI-1640 medium containing 10% FBS. The cells were washed once in serum-free medium and resuspended in PBS. Suspensions consisting of Panc-1 cells, with >90% viability, were used for the injections.

### Cell Survival Rate Detected by CCK-8

HPNE, ECs, SW1990 cells, and Panc-1 cells collected in exponential phase were seeded at an initial density of 4×10^3^ cells per well in 96-well plates. Then cells were divided into four groups with different treatment for 72 h: the control group (0.1% DMSO), the gemcitabine group (gemcitabine, 20 µmol/L), the emodin group (emodin, 40 µmol/L), and the combination group (gemcitabine 20 µmol/L + emodin 40 µmol/L), with five wells for each group. The wells without drugs but 0.1% DMSO were used as control. One hour before the exposure ended, 0.01 mL CCK-8 was added into each well. The plates were mixed for 10 min on a gyratory shaker, and absorbance at the 450 nm wavelength was measured with an ELISA reader (BIO-Tek ELx800, Winooski, VT, USA). The experiment was repeated three times. The cell survive rate was calculated as follows: cell survive rate  =  (experimental OD value - blank OD value)/(control OD value - blank OD value) × 100%.

### Flow Cytometric Assessment of Apoptosis

SW1990 cells and Panc-1 cells at the density of 2×10^5^/ml were cultured overnight in six-well plates, and at 80% confluence, they were treated in triplicate with 20 µmol/L gemcitabine, 40 µmol/L emodin, and their combination, respectively, for 72 h. The cells without drug treatment were used as control. Subsequently, the cells were harvested, washed, and stained with 10 µL Annexin V and 5 µL propidium iodide (PI) in the dark at room temperature for 15 min, according to the manufacturer’s instruction (Biosea, Beijing, China). The apoptotic cells were determined using flow cytometer (Epics AltraII, Beckman Coulter, USA) and examined under an inverted fluorescent microscope.

### In vitro Angiogenesis Assay

Analysis of capillary formation was performed using an *in vitro* angiogenesis kit (Chemicon, Temecula, CA) according to the manufacturer’s instructions. Briefly, 50 µl of gel matrix solution was applied into one well of a 96-well plate and incubated at 37°C for 1 h. ECs isolated from the implanted pancreatic cancer tissues were then trypsinized. 5×10^3^ cells were suspended in 50 µl RPMI-1640 culture medium containing various concentrations of VEGF, plated onto the gel matrix, and incubated in a 5% CO2-humidified atmosphere at 37°C for 24 h. The cell three-dimensional organization was examined under an inverted photomicroscope using following grades [27]: 0, individual cells, well separated; 1, cells begin to migrate and align themselves; 2, capillary tubes visible, no sprouting; 3, sprouting of new capillary tubes visible; 4, closed polygons begin to form; and 5, complex mesh-like structures develop. A blinded investigator examined 10 randomly selected fields. Then“Angiogenic index”was got from calculating the average grade examined as above. Each treatment was performed in triplicate.

### Western Blot Analysis Detecting Apoptosis-related Proteins

SW1990 cells and Panc-1 cells at the density of 2×10^6^/plate were treated with drug(s) as described above and harvested. After cells were washed, nuclear proteins were extracted and the protein concentration was determined using the BCA assay. the cells were lysed in 200 µl ice-cold lysis buffer (300 mmol/l NaCl, 50 mmol/l Tris–HCl (pH7.6), 0.5% TritonX-100, 2 mmol/l phenylmethanesulfonyl fluoride, 2 µl/ml aprotinin, and 2 µl/ml leupeptin). The protein concentration in the lysates was determined using a BCA protein assay kit (Pierce Rockford, Illinois, USA), according to the manufacturer’s instruction. The lysates (20 µg/lane) were separated using 8–12% sodium dodecylsulfate–polyacrylamide gel (SDS-PAGE) electrophoresis and transferred onto polyvinylidene fluoride (PVF) membranes. After being blocked with 5% fat-free milk, the membranes were probed with individual primary antibodies overnight at 4°C and the bound antibodies were detected with horseradish peroxidase (HRP)-conjugated goat anti-rabbit IgG. The formed immunocomplex was visualized using enhanced chemiluminescence reagent and exposed to X-ray films.

### Orthotopic Implantation of Pancreatic Cancer and Treatment Protocol (Fig.4A)

Briefly, 2×10^6^ pancreatic cancer Panc-1 cells were subcutaneously injected into the flanks of donor nude mice. After reaching a volume of ∼1 cm^3^, subcutaneous tumors were removed under sterile conditions. The central necrotic tissues in the tumor were cleared and the healthy peripheral tissues of the subcutaneous tumor were cut into 1 mm^3^ tissue blocks for orthotopic transplantation. 48 recipient nude mice were anesthetized with pelltobarbitalum natricum (50 mg/kg) and opened by a left longitudinal laparotomy. The spleen, together with the pancreatic tail, was gently exteriorized, and a tissue pocket in the pancreatic parenchyma was created by microscissors. One tumor fragment was placed into the tissue pocket in a way that it was entirely surrounded by normal pancreas. After careful relocation of the pancreas and spleen into the abdominal cavity, the animals were closed in 2 layers for the muscular layer and nonabsorbable stainless steel wound clips for the skin.

Two weeks after the *in situ* xenografts model of pancreatic cancer was built, the nude mice were randomly divided into four groups with 12 mice in each group: the control group, in which 0.2 mL 0.9% sodium chloride was injected into the abdominal cavity of each mouse; the gemcitabine group, in which gemcitabine (100 mg/kg) was injected in the same way; the emodin group, in which emodin (50 mg/kg) was perfused into the stomach for each mouse; the combination group, in which mice were give gemcitabine (80 mg/kg) through intraperitoneal injection and emodin (50 mg/kg) through intragastric administration. Each group was treated with drug 3 times per week for 2 weeks. Mice bearing orthotopically implanted tumors were imaged by MicroPET for ^18^F-FDG uptake (Detailed method is described below) 4 weeks after the drug treatment were completed. Mice were sacrificed 8 weeks after implantation and then pancreatic tumors were carefully separated and weighed. Part of the orthotopic tumor tissues was stored in 4% paraformaldehyde for immunohistochemistry, and the remainder of the specimens was frozen in liquid nitrogen.

### MicroPET Imaging

Mice were fasted for at least 8 h before imaging, and 0.1 mCi ^18^F-FDG/mouse was injected into the tail vein. Mice were anesthetized using 2% isoflurane, positioned in a prone position along the long axis of the microPET scanner, and imaged. A 10 min data collection was performed in ^18^F-FDG-PET with an uptake time of 1 h after the tracer injection. Static acquisition was performed in three-dimensional mode using a microPET imaging system (R4, Concorde Microsystems, Knoxville, TN, USA). For quantitative evaluation, the regions of interest (ROIs) method and standard uptake value (SUV) were used to evaluate the regional uptake of the tracers. MicroPET ASIPro6.0.5.0 (Concorde Microsystems, Inc., Knoxville, TN) was used for statistical analysis of ROIs and SUVs. ROIs were drawn around the tumors and surrounding areas on the coronal slices that showed the best delineation of the tumors. All images were displayed on the same color scale. The highest uptake (ratio to the injection dose) within the tumor ROI and the mean uptake (ratio to the injection dose) in the surrounding ROI was recorded and calculated. T/N ratio was calculated for semiquantitative analysis using the following formula: T/N ratio  =  pixel maximum uptake in tumor ROI/pixel mean uptake in surrounding ROI. T/N ratio stands for tumor growth metabolism. The SUVs for ROIs at different groups were compared. SUV  =  concentration of radioactivity in the ROI (mCi/ml) × body weight (g)/injected dose (mCi).

### Tumor Vascular Imaging

Based on the same model establishing protocol as above, 4 weeks after the drug treatment were completed, orthotopically implanted tumors in mice were imaged using the vascular imaging approach**.** After tumor-bearing nude mice were intraperitoneally injected with pentobarbital sodium (50 mg/kg), a ∼1.5 cm incision was made in the middle of the chest. The chest was opened to clearly expose the heart. Catheters were inserted into the aortic arch from heart apex. Then imaging agent were perfused through the catheters at the dose of ∼20 ml/kg. The nude mice were then stored at −20°C for 24 h. The tumors were separated after the nude mice were thawed. In the end, the vascular images of the tumors were captured using an X-ray machine (Shanghai Medical Instruments Corporation, GX-211) at a current of 100 mA and a voltage of 70 kV. The computed radiography scan imaging system (CR) and the image workstation (Foxconn, FCG XG-1) were used. Image analysis was performed using Scion Image Beta 4.02 software (Scion Corporation, Frederick, MD, USA). The vascular density was calculated as the total blood vessel volume in tumor divided by the tumor volume.

### Immunohistochemistry Detecting ki-67 and Angiogenesis-associated Protein: CD31 (PECAM-1), CD105 (Endoglin), NF-κB, MMP-2, MMP-9, VEGF and eNOS

Sections were cut from paraffin embedded pancreatic cancer tissues. Sections were blocked with goat serum and immunostained after deparaffinization and rehydration. Immunostaining was performed using primary antibodies specific for ki-67, NF-κB, MMP-2, MMP-9, VEGF, and eNOS with appropriate dilutions, followed by staining with appropriate HRP-conjugated secondary antibodies. The slides were developed in diaminobenzidine and counterstained with a weak solution of haematoxylin solution stain. The stained slides were dehydrated and mounted in permount and visualized on a microscope (Olympus, Japan). Images were captured with an attached camera linked to a computer. For data quantification, the integrated optical density (IOD) was analyzed using Image-Pro Plus 6.0 software. Twelve nude mice were included for each experimental group. One section was obtained from each animal. At least six fields were randomly selected from each section. The average IOD levels in different groups were then compared.

**Table 1:** Primer pairs used in reverse transcription polymerase chain reaction.

Genes	Primers (5′-3′)	Product length (bp)
*NF-kB*	Forward CCTGCACTCAATCAAGAAGTTGC	300
	Reverse TTCCTGCTCTGTTTGGTGAGGCT	
*VEGF*	Forward TGCCCACTGAGGAGTCCAAC	336
	Reverse TGGTTCCCGAAACGCTGAG	
*MMP-2*	Forward GAGAACCAAAGTCTGAAGAGCGTCAA	397
	Reverse TGTGAAAGGAGAAGAGCCTGAAGTG	
*MMP-9*	Forward TCAGGGAGACGCCCATTT	440
	Reverse TCGGGCAGAAGCCGAAG	
*eNOS*	Forward CAGTGTCCAACATGCTGCTGGAAATTG	485
	Reverse TAAAGGAGGTCTTCTTCCTGGTGATGCC	
*GAPDH*	Forward AACGGATTTGGTCGTATTGGG	336
	Reverse TCGCTCCTGGAAGATGGTGAT	

### Electrophoretic Mobility Shift Assay (EMSA)

NF-κB activity was evaluated using the EMSA analysis. Nuclear proteins were extracted from pancreatic cells or implanted pancreatic cancer tissues and the protein concentration was determined usng the BCA assay. The Biotin end-labeled DNA duplex of sequence 5′-AGT TGAGGG GAC TTT CCC AGG C-3′ and 3′-TCA ACT CCC CTG AAA GGG TCC G-5′ containing a putative binding site for NF-κB was incubated with the nuclear extracts. Subsequently, the DNA-protein complexes were subjected to a 6% native polyacrylamide gel electrophoresis and transferred to a nylon membrane (Pierce), and cross-linked on a UV transilluminator for 15 min, followed by detection using the LightShift™ Chemiluminescent EMSA kit (Pierce), according to the manufacturers’ instruction. The membranes were exposed to X-ray films and the relative intensities were analyzed using an NIH Image 1.62 package. The nuclear extracts from gastric cancer SGC7901 were used as negative control, and the cells stimulated with 50 ng/ml TNFα were used as positive control.

### Western Blot Analysis Detecting Angiogenesis-associated Protein: NF-κB, MMP-2, MMP-9, VEGF and eNOS

SW1990 cells and Panc-1 cells at a density of 2×10^6^/plate were treated with drug(s) as described above and harvested. Nuclear proteins were extracted from SW1990 cells, Panc-1 cells, and tumor tissues for NF-κB detection, and then the concentration was determined using the BCA assay. For detection of other proteins, the total proteins were routinely extracted from tumor tissues using RIPA lysis buffer. The protein concentration was determined using the BCA assay. The proteins were then fractionated using SDS-PAGE, electro-transferred to PVDF membranes, blocked with 5% non-fat milk, and then probed with primary antibodies and HRP-conjugated anti-rabbit secondary antibody. After washing, the bound antibody complexes were analyzed using an ECL chemiluminescence reagent (Amersham, USA).

### RT-PCR Detected mRNA Levels of NF-κB, VEGF, MMP-2, MMP-9 and eNOS

Total RNA in tumor tissues was extracted using TRIzol reagent according to the manufacturer's instructions, and purified using an RNAfast-200 purification kit. The RNA was quantified using the spectrophotometric analysis, and the RNA integrity was verified using agarose gel electrophoresis. The cDNA was synthesized with the iScript cDNA synthesis kit (Bio-Rad) using 2 µg RNA. The cDNA product was diluted 1:50 in deionized water, and primers specific for NF-κB, VEGF, MMP-2, MMP-9 and eNOS and the GAPDH control were adjusted to 10 pmol/µl working stock with deionized water. Reverse transcription-polymerase chain reaction (RT-PCR) was conducted with the diluted cDNA and primers using an ABI Prism 7700 (Applied Biosystems, Foster City, CA, USA) following the protocol outlined in the iTag SYRB Green Supermix with ROX kit (Bio-Rad). The amplification reaction was carried out with 1 µl cDNA product for 30 cycles. The RT-PCR products were visualized on 2% agarose gels with ethidiumbromide staining under UV transillumination. The band intensity was analyzed using QuantityOne version 4.5 software (Bio-Rad). The primers used are listed in Table 1.

### Statistical Analysis

Data shown are representative images or expressed as mean ± SEM of each group. The difference among groups of cells or mice was analyzed using the chi-square test or ANOVA, followed by post hoc Student’s t-test using the SPSS17.0 software. *P*<0.05 was considered statistically significant.

## References

[1] Louvet C, Labianca R, Hammel P, Lledo G, Zampino MG, et al (2005) Gemcitabine in combination with oxaliplatin compared with gemcitabine alone in locally advanced or metastatic pancreatic cancer: results of a GERCOR and GISCAD phase III trial. J Clin Oncol 23: 3509–3516. 15908661 10.1200/JCO.2005.06.023

[2] Abou-Alfa GK, Letourneau R, Harker G, Modiano M, Hurwitz H, et al (2006) Randomized phase III study of exatecan and gemcitabine compared with gemcitabine alone in untreated advanced pancreatic cancer. J Clin Oncol 24: 4441–4447. 16983112 10.1200/JCO.2006.07.0201

[3] Gately S, Kerbel R (2003) Therapeutic potential of selective cyclooxygenase-2 inhibitors in the management of tumor angiogenesis. Prog Exp Tumor Res 37: 179–192. 12795055 10.1159/000071373

[4] North S, Moenner M, Bikfalvi A (2005) Recent developments in the regulation of the angiogenic switch by cellular stress factors in tumors. Cancer Lett 218: 1–14. 15639335 10.1016/j.canlet.2004.08.007

[5] Kunnumakkara AB, Guha S, Krishnan S, Diagaradjane P, Gelovani J, et al (2007) Curcumin potentiates antitumor activity of gemcitabine in an orthotopic model of pancreatic cancer through suppression of proliferation, angiogenesis, and inhibition of nuclear factor-kappaB-regulated gene products. Cancer Res 67: 3853–3861. 17440100 10.1158/0008-5472.CAN-06-4257

[6] Liu A, Chen H, Tong H, Ye S, Qiu M, et al (2011) Emodin potentiates the antitumor effects of gemcitabine in pancreatic cancer cells via inhibition of nuclear factor-kappaB. Mol Med Report 4: 221–227. 10.3892/mmr.2011.414 21468555

[7] Wang ZH, Chen H, Guo HC, Tong HF, Liu JX, et al (2011) Enhanced antitumor efficacy by the combination of emodin and gemcitabine against human pancreatic cancer cells via downregulation of the expression of XIAP in vitro and in vivo. Int J Oncol 39: 1123–1131. 21743963 10.3892/ijo.2011.1115

[8] Chen H, Wei W, Guo Y, Liu A, Tong H, et al (2011) Enhanced effect of gemcitabine by emodin against pancreatic cancer in vivo via cytochrome C-regulated apoptosis. Oncol Rep 25: 1253–1261. 21305255 10.3892/or.2011.1174

[9] Kaneshiro T, Morioka T, Inamine M, Kinjo T, Arakaki J, et al (2006) Anthraquinone derivative emodin inhibits tumor-associated angiogenesis through inhibition of extracellular signal-regulated kinase 1/2 phosphorylation. Eur J Pharmacol 553: 46–53. 17056031 10.1016/j.ejphar.2006.09.026

[10] Kwak HJ, Park MJ, Park CM, Moon SI, Yoo DH, et al (2006) Emodin inhibits vascular endothelial growth factor-A-induced angiogenesis by blocking receptor-2 (KDR/Flk-1) phosphorylation. Int J Cancer 118: 2711–2720. 16388516 10.1002/ijc.21641

[11] Lu Y, Zhang J, Qian J (2008) The effect of emodin on VEGF receptors in human colon cancer cells. Cancer Biother Radiopharm 23: 222–228. 18454691 10.1089/cbr.2007.0425

[12] Gonzalez-Perez RR, Xu Y, Guo S, Watters A, Zhou W, et al (2010) Leptin upregulates VEGF in breast cancer via canonic and non-canonical signalling pathways and NFkappaB/HIF-1alpha activation. Cell Signal 22: 1350–1362. 20466060 10.1016/j.cellsig.2010.05.003 PMC2928711

[13] Banerjee S, Zhang Y, Ali S, Bhuiyan M, Wang Z, et al (2005) Molecular evidence for increased antitumor activity of gemcitabine by genistein in vitro and in vivo using an orthotopic model of pancreatic cancer. Cancer Res 65: 9064–9072. 16204081 10.1158/0008-5472.CAN-05-1330

[14] Crawford AD, Liekens S, Kamuhabwa AR, Maes J, Munck S, et al (2011) Zebrafish bioassay-guided natural product discovery: isolation of angiogenesis inhibitors from East African medicinal plants. PLoS ONE 6: e14694. 21379387 10.1371/journal.pone.0014694 PMC3040759

[15] Carbone C, Melisi D (2012) NF-kappaB as a target for pancreatic cancer therapy. Expert Opin Ther Targets 16 Suppl 2 S1–10. 10.1517/14728222.2011.645806 22443181

[16] Meng F, Henson R, Patel T (2007) Chemotherapeutic stress selectively activates NF-kappa B-dependent AKT and VEGF expression in liver cancer-derived endothelial cells. Am J Physiol Cell Physiol 293: C749–760. 17537803 10.1152/ajpcell.00537.2006

[17] Babykutty S, P SP, R JN, Kumar MA, Nair MS, et al (2011) Nimbolide retards tumor cell migration, invasion, and angiogenesis by downregulating MMP-2/9 expression via inhibiting ERK1/2 and reducing DNA-binding activity of NF-kappaB in colon cancer cells. Mol Carcinogen 51: 475–490. 10.1002/mc.20812 21678498

[18] Ye Y, Martinez JD, Perez-Polo RJ, Lin Y, Uretsky BF, et al (2008) The role of eNOS, iNOS, and NF-kappaB in upregulation and activation of cyclooxygenase-2 and infarct size reduction by atorvastatin. Am J Physiol Heart Circ Physiol 295: H343–351. 18469150 10.1152/ajpheart.01350.2007

[19] Liu H, Chen A, Guo F, Yuan L (2010) A short-hairpin RNA targeting osteopontin downregulates MMP-2 and MMP-9 expressions in prostate cancer PC-3 cells. Cancer Lett 295: 27–37. 20207476 10.1016/j.canlet.2010.02.012

[20] Liu A, Chen H, Wei W, Ye S, Liao W, et al (2011) Antiproliferative and antimetastatic effects of emodin on human pancreatic cancer. Oncol Rep 26: 81–89. 21491088 10.3892/or.2011.1257

[21] Ferrara N, Alitalo K (1999) Clinical applications of angiogenic growth factors and their inhibitors. Nat Med 5: 1359–1364. 10581076 10.1038/70928

[22] Yin JH, Shi WD, Zhu XY, Chen Z, Liu LM (2011) Qingyihuaji Formula Inhibits Progress of Liver Metastases From Advanced Pancreatic Cancer Xenograft by Targeting to Decrease Expression of Cyr61 and VEGF. Integr Cancer Ther 11: 37–47. 21382954 10.1177/1534735411400315

[23] Aggarwal BB (2004) Nuclear factor-kappaB: the enemy within. Cancer Cell 6: 203–208. 15380510 10.1016/j.ccr.2004.09.003

[24] Lu HF, Lai KC, Hsu SC, Lin HJ, Kuo CL, et al (2009) Involvement of matrix metalloproteinases on the inhibition of cells invasion and migration by emodin in human neuroblastoma SH-SY5Y cells. Neurochem Res 34: 1575–1583. 19291397 10.1007/s11064-009-9946-3

[25] Chen TG, Chen JZ, Wang XX (2006) Effects of rapamycin on number activity and eNOS of endothelial progenitor cells from peripheral blood. Cell Prolif 39: 117–125. 16542347 10.1111/j.1365-2184.2006.00375.x PMC6495845

[26] Papapetropoulos A, Garcia-Cardena G, Madri JA, Sessa WC (1997) Nitric oxide production contributes to the angiogenic properties of vascular endothelial growth factor in human endothelial cells. J Clin Invest 100: 3131–3139. 9399960 10.1172/JCI119868 PMC508526

[27] Bahlmann FH, DeGroot K, Duckert T, Niemczyk E, Bahlmann E, et al (2003) Endothelial progenitor cell proliferation and differentiation is regulated by erythropoietin. Kidney Int 64: 1648–1652. 14531796 10.1046/j.1523-1755.2003.00279.x

